# Fuel cells are a commercially viable alternative for the production of “clean” energy

**DOI:** 10.1007/s13280-015-0731-z

**Published:** 2015-12-14

**Authors:** Dimitris K. Niakolas, Maria Daletou, Stylianos G. Neophytides, Constantinos G. Vayenas

**Affiliations:** Foundation for Research and Technology, Institute of Chemical Engineering Sciences (FORTH/ICE-HT), Stadiou str., 26504 Platani, Rion Patras Greece; Department of Chemical Engineering, LCEP, University of Patras, Patras, Greece

**Keywords:** Fuel cells, Market position, Policy making, Sustainable energy, Technology level

## Abstract

Fuel cells present a highly efficient and environmentally friendly alternative technology for decentralized energy production. The scope of the present study is to provide an overview of the technological and commercialization readiness level of fuel cells. Specifically, there is a brief description of their general advantages and weaknesses in correlation with various technological actions and political strategies, which are adopted towards their proper positioning in the global market. Some of the most important key performance indicators are also discussed, alongside with a few examples of broad commercialization. It is concluded that the increasing number of companies which utilize and invest on this technology, in combination with the supply chain improvements and the concomitant technological maturity and recognition, reinforce the fuel cell industry so as to become well-aligned for global success.

## Introduction

The European Union is committed to transforming its transport and energy systems into low-carbon systems by 2050 and to decouple economic growth from resource and energy use, reducing greenhouse gas emissions, increasing energy security, while maintaining a strong competitive global position. Some recent studies (Lund 2010; Akikur et al. 2014; Maalej et al. 2014; Rothuizen and Rokni 2014; Yazdanie et al. 2014) have concluded that hydrogen, together with electricity, alternative power sources, sustainable biofuels and natural gas, could gradually become a much more significant component of the European energy mix. Fuel cells at the same time are the most efficient means of converting various fuels, especially hydrogen, to clean, efficient, reliable power and heat for a wide range of energy-related applications, including portable devices, combined heat and power (CHP) and road and non-road transport (FCH JU-2-MAWP 2014).

Fuel cells (FC) and hydrogen (H_2_) systems offer a potential long-term energy option, but still face some major challenges in facilitating their market breakthrough, despite the significant progress that has been achieved the last 20 years. Being a commercially embryonic technology compared to traditional energy sources, the resources needed to bring fuel cells into a commercial breakthrough necessary for a fast market penetration are still significant (Lund 2010). It is a common belief that nowadays, we are witnessing the beginning of an extremely exciting time for fuel cell and hydrogen technologies, driven primarily by three forces: the recognition of hydrogen as an attractive and important energy storage platform by energy utilities; the interest of major global telecoms in fuel cell backup power; and the commercialization of fuel cell electric vehicles (FCEV) by the world’s major automakers. It is a fact that an early market has already evolved. Small fuel cells for charging of smartphones are already commercially available (i.e. PowerTrekk^1^), as well as small CHP units where more than 100 000 units have been deployed in the Japanese ENE‐FARM program. Another successful example is the installation of more than 100 MW of MCFC units for distributed CHP in the US and South Korea. These are some early applications, but of great importance, towards the further growth of the fuel cells and hydrogen field. With an increasing number of truly global companies utilizing and investing in fuel cell technology and with the supply chain improvements and technology recognition that will come with the mass manufacture of passenger vehicles, the fuel cell industry is becoming increasingly well-aligned for global success.

Consequently, fuel cells and hydrogen are part of the portfolio of technologies identified in the strategic energy technology (SET) Plan with expected contributions to a sustainable and secure energy supply system in the medium and, mainly, in the long-term. This is also consistent with the goals of the EU 2020 strategy, the energy 2050 roadmap, the white paper on transport, the communication on research and innovation for Europe’s future mobility (strategic transport technology plan, STTP) and the communication on clean power for transport outlining the European alternative fuels strategy (FCH JU-2-MAWP 2014).

Fuel cells are basically open thermodynamic systems. They operate on the basis of electrochemical reactions and consume reactant from an external source (Mekhilefa et al. 2012). They are favourable alternatives to conventional electricity generation methods for small-scale applications. Hydrogen and hydrocarbon fuels contain significant chemical energy in comparison with conventional battery materials; hence they are now widely developed for numerous energy applications (Mekhilefa et al. 2012). Fuel cell technology is a promising substitute for fossil fuels to provide energy for rural areas where there is no access to the public grid or huge cost of wiring and transferring electricity is required. In addition, applications with essential secure electrical energy requirement such as uninterruptible power supplies (UPS), power generation stations and distributed systems can employ fuel cells as their device for secure energy production (Mekhilefa et al. 2012).

In general, fuel cells are different according to their operating temperature, efficiency, applications and costs. They are classified based on the choice of fuel and electrolyte into six major groups (Mekhilefa et al. 2012):

- Alkaline fuel cell (AFC)
- Phosphoric acid fuel cell (PAFC)
- Solid oxide fuel cell (SOFC)
- Molten carbonate fuel cell (MCFC)
- Proton exchange membrane fuel cell (PEMFC), including the subcategory of direct methanol fuel cells (DMFCs)

PEM fuel cell technology has proven to be the most popular type of fuel cell with regard to unit shipments. This can be attributed to its wide application across all applications, unlike other fuel cell types, and its suitability for use at both small and large scales. In terms of delivered megawatts per fuel cell type and according to a recent report on the industrial development from E4tech (2014) MCFCs were dominating in 2013, the PEMFCs were second and the SOFCs were third in the ranking producing about half of that of MCFCs.

Due to the increase in market pull and the number of products available, the residential combined heat and power (resCHP) market is experiencing annual double-digit growth. The small, stationary fuel cells used for resCHP systems typically range in power ratings from 0.8 kW to up to 8 kW and provide on-site electricity and low-grade heat generation for the home.

Finally, comparison of the estimated capital costs between ICEVs (internal combustion electric vehicles) and FCEVs (fuel cell electric vehicles) shows that although the latter is more expensive due to costs involved with hydrogen system modifications and distribution infrastructure, the operational costs during the vehicle’s lifetime are less. Current innovative and modern fuel cell technologies need to meet the economical features and exceed the advantages of the existing technologies to be acceptable for mass production. In order to improve the feasibility and to increase the efficiency of FCEVs, more R&D should be conducted by research institutes and industries. Fuel cells offer a number of important advantages over internal combustion engine (ICE) and other current power generator systems (Mekhilefa et al. 2012).

## General strengths of the fuel cell technology systems

The major advantage of fuel cells is their high thermodynamic efficiency, which can take realistic values in the range of 40–60 %. Meanwhile, there is concomitant production of heat with the electric energy; heat that is available at slightly lower temperature of the one that the cell operates. This means that fuel cells have the potential to be used for cogeneration of electricity and heat, covering thus the heat and power needs for domestic and other larger scale industrial applications, which is very interesting under the perspective of the steadily increased tendency for decentralized power production.

Fuel cell systems are flexible regarding the power output and they can be used for the power production of electrical power in the region from 50 W to 100 MW. Specifically, the power output of small portable systems can be as low as a few watts, whereas in the case of biological fuel cells for medical applications the power output can be lower. In contrast to the conventional heating systems, they possess apart from the highest thermodynamic output factor, the following two advantages: (i) the efficiency for the electrical energy production is preserved in high values even for small-scale units, while its value is high under partial load conditions and it can be higher than that under full load conditions and (ii) this technology is environmental friendly, taking into consideration that the pollution emissions are negligible when using H_2_.

Especially in the case where hydrogen is the main fuel there are no polluting emissions at all, while in the case of other fuels, such as natural gas, the quantity of the polluting emissions is approximately two orders of magnitude lower than that in the case of the conventional electro-productive systems.

## General weaknesses of the fuel cells systems

- Durability issues/stability and useful lifetime
- Major challenges in producing, transporting and storing hydrogen
- Production cost use of (rare-noble) expensive raw elements and materials.

For example, the catalyst being extensively used in PEFMCs is Platinum (Pt). Consequently, if someone considers the possible scenario in which all vehicles are powered by PEMFCs, then it is most likely that the world reserves of this precious metal are not enough for the future needs. Therefore, there is extensive research activity for the decrease of Pt content or for its replacement with other cheaper and if possible more efficient materials.

In the SOFC category that can also operate with natural gas as the fuel a major problem comes with the deactivation/poisoning of the fuel cell electrodes, which is attributed to carbon deposition and/or sulphur poisoning. This deactivation has a negative impact on the stability and reliability of the whole system, while it constitutes another worldwide research field of intensive activities, for the development of effective and tolerant materials.

Furthermore, the controlling procedures of many (full power) units are time consuming and expensive. These processes are imperative for ensuring that the reliability and the lifetime of the fuel cell systems will last for approximately 5 000 and 40 000 h for portable and stationary applications, respectively, in order to be commercially viable.

Finally, H_2_ infrastructure for fuelling or even the on-site production of H_2_ has to be developed. This can be accomplished by the parallel development of renewable H_2_ production and storage technologies either on site or through a distributed network. The main H_2_ production technologies comprise water electrolysis from renewable electricity and biofuels production and reforming. The cost of production, transportation and storage of H_2_ is still high and needs to be tackled.

Regarding the adopted actions and strategies in a global extent, the United Nations Environmental programme envisages 27 GW of installed fuel cell capacity in OECD Europe for the year 2020. Moreover, the US total investment in fuel cell companies in 2012 was $307.1 million, while the US Department of Energy (DOE) is continuing to show support for next-generation fuel cell systems. In June 2013, DOE rolled out $9 million in grants to speed up the technology, while $4.5 million will be invested in two projects focused on advanced fuel cell membranes (Minnesota based-3 M and Colorado School of Mines). These projects fall in line with other DOE projects during the past decade with the goal of improving efficiency and lowering costs for fuel cells. The research projects have helped cut down on the amount of platinum used in catalysts by a factor of five. They have also reduced the costs of transportation with fuel cells more than 80 % since 2002.

Asia and especially Japan and South Korea constitute the leading market for fuel cells and is likely the most dynamic region for fuel cell development right now. Specifically, Asia continues to dominate the fuel cell industry in terms of system shipments with 28 000 in 2012 and 51 100 in 2013, which correspond to 61 and 75 %, respectively, of the global market. More precisely, the former category is dominated by Japan with more than 40 000 residential CHP units, likely to be shipped during 2014, and several thousand units for backup power installed throughout Asia. Furthermore, Asia overtook North America to lead the 2012 megawatt count with 86.1 MW, or 52 % of the total. North America followed second with 37 % in 2012, while Europe was third with approximately 10 % in 2012. It is noteworthy to be mentioned that this ranking continues in 2013 and in the forecasts for 2014 (FUELCELLTODAY 2013; E4tech 2014). Particularly for the MW field, Korea remains the leading market, mostly due to the ongoing installation of large fuel cell systems for prime power in dedicated fuel cell parks.

On the other hand, the European Commission has supported research and development in fuel cells and hydrogen technologies since the early EU Framework Programmes (FP) with increasing funding levels over time (e.g. 145 million € in FP5, 315 million € in FP6) (FCH JU-2-MAWP 2014).

In May 2008 the Council adopted a Regulation (EC no. 521/2008) setting up a Joint Undertaking for the implementation of the Joint Technology Initiative on Fuels Cells and Hydrogen (hereinafter referred to as “FCH JU”) on the basis of Article 171 of the EC Treaty, now replaced by Article 187 of the TFEU (Treaty on the Functioning of the European Union) (FCH JU-2-MAWP 2014).

The main target is that by 2020, fuel cell and hydrogen technologies should be demonstrated as one of the pillars of future European energy and transport systems, making a valued contribution to the goals of the European strategic energy technology plan (SET Plan) (European Commission 2007, p. 723) and the European strategic transport technology plan (STTP) (SWD (2012) 260 final), contributing significantly to the transformation to a low-carbon economy by 2050. The overall commitment of stakeholders remains strong as shown by a recent survey performed by the FCH JU (FCH JU), even if the economic crisis and the tendency to reduce investment in longer term research have affected some strong industry and research players.

Some of the main objectives that need to be achieved by 2020, in order to ensure that the performances of the technologies will allow for their progressive deployment and their full integration in a low-carbon economy during the period up to 2050 (FCH JU-2-MAWP 2014) are as follows:

- Reducing by a factor of 10 the production cost of fuel cell systems to be used in *transport applications* (currently 500 €/kW for cars) thanks to scientific and technology progress as well as scale effects when series production is launched—while increasing lifetime by a factor of 2 (currently 2 500 h for cars).
- Increasing the electrical efficiency of fuel cells for power production by on average 10 percentage points (currently 40–50%), while reducing cost by a factor of 3 (currently 4 500–8 000 €/kW) and increasing the durability by a factor of 4 (currently 8 000–15 000 h for PEMFCs, 40 000–50 000 h for SOFCs and 70 000–80 000 h for MCFCs).
- Increasing the energy efficiency of hydrogen production via electrolysis from 67 to 77 % while reducing the investment cost to below 2 million€/t per day capacity (currently 3–4 million€/t).
- Demonstrating at large scale (10’s to 100 MW) the feasibility of hydrogen as a competitive energy storage medium for integration of electricity produced from renewable primary energy.

## Proposed research directions on materials, processes and fuel cell systems

The new multiannual working plan (MAWP) of the FCH JU is focussing its activities on demonstration and field testing projects aiming to the faster development of fuel cell systems and their market penetration. However, the massive use of fuel cells still needs breakthrough research on materials and their interfaces (mainly the electrochemical interfaces) as these are summarised in the following topics:

- Novel materials and novel fuel cell design concepts, which will allow the effective reduction of precious metal loadings. This can be achieved through two main approaches. One is the development of novel, more active, electrocatalysts aiming to atomic distribution of the metal active phase on stable nanostructured supporting materials. The other approach is through the synthesis and development of stable anionic alkaline polymer electrolytes, which will allow the use of non precious metal electrocatalysts.
- Simulation and understanding the functionality and operational characteristics of a 3D structured electrochemical interface. This research topic aims to (i) 100 % utilization of the active electrocatalyst and (ii) the innovative design of the flow fields. As a result, a uniform distribution of the reacting gases can be achieved along the 3D structure of the catalytic layer, i.e. below the gas streams and below the ribs of the bipolar plates.
- Novel designs, engineering and operational concepts can be conceived so as to improve the performance of fuel cells. This advance can be accomplished by means of an integrated approach, based both on materials development and on the deployment of innovative cell designs. The specific strategy will permit the effective control of (i) the electrocatalytic activity, especially in terms of the efficiency of the electrochemical interfaces and (ii) the poisoning effect of the feeding gases on the electrodes’ performance.

Regarding the fuel cell systems development, the MAWP of FCH JU is focusing its activities for the next seven years on the development of fuel cell systems for transportation by the use of pure H_2_ and CHP units for stationary applications, operating on natural gas and/or hydrocarbons reforming. Nevertheless, on the basis of future systems development towards 2050, fuel cell systems can play the leading role in a sustainable decentralized and environmentally being H_2_ economy, as well as in the area of wastewater purification and electricity production. A brief description of such systems is given below:

- Regenerative or unitized fuel cells can be developed as energy storage/production devices where renewable energy can be stored in a closed water loop in the form of H_2_ by water splitting through electrolysis, while fuel cell can consume stored H_2_ at will for the production of electricity. An advanced version of such a system is the development of a unitized fuel cell/electrolyser in one device (Millet et al. 2011). The technological feasibility and challenge of such a system depends on the development of anode materials, which can be used in polymer electrolyte membrane systems both as anodes for fuel cells and water electrolysis. Furthermore, the same technological approach can be achieved through the development of high temperature Solid Oxide Electrolysis (SOECs) and Molten Carbonate Electrolysis Cells (MCECs). These systems show great dynamics to become commercially competitive against other electrolysis technologies (AEL, PEMEL), which are better established in the market but more expensive and less efficient (Grindler et al. 2014).
- Photo excited electrochemical devices can be used for the photo oxidation of organic load in wastewater and electricity cogeneration. The process is based on the photoelectroreforming of the organic matter on n-type semiconducting photoanodes and the production of protons H^+^ which can readily reduce O_2_ at the cathode for the sustainable electricity production. This process can be termed as “photo fuel cell” and the energy that can be produced by the efficient photoinduced mineralization of urban wastes can be up to 10 % of the electricity of the urban area.

## Conclusion

Fuel Cells and Hydrogen (FCH) technologies introduce radical changes and their potential social and environmental benefits will not be monetized on the short term, which increases the investment risk for early movers. Despite its significant progress, the technology has not yet achieved competitive levels of life-cycle cost and overall performance required for a large-scale deployment, though the commercialization of some specific products (e.g. passenger cars, buses, materials handling vehicles, backup power, portable power) has already begun. Thus, a strong technology leapfrogging to reduce the costs would be very beneficial. Large concerted research and development (R&D) actions such as the European fuel cell and hydrogen joint technology effort, the Japanese ENE-FARM program and other actions would be highly justified and could save billions of euros in market deployment efforts otherwise required. The time to breakthrough could similarly be reduced by 60–70 % (Lund 2010). On the other hand, it has to be mentioned that so far all fuel cells that have been commercially introduced are, more or less, based on “conventional” materials and concepts, which have been tested and technologically established for quite some time. Thus, a large-scale introduction of fuel cells might be already feasible even without major breakthroughs. The latter statement is further verified by the recent commercial introduction of fuel cell vehicles by Toyota and Hyundai.

The great opportunity for fuel cells is issued from the fact that this technology connects two basic future energy carriers: electricity and hydrogen. Fuel cells are the most appropriate technology for the conversion of the hydrogen`s chemical energy to electricity, due to the high conversion efficiency. Furthermore, it is a common belief that we are witnessing the beginning of an extremely exciting time for fuel cell and hydrogen technologies, driven primarily by three forces: the recognition of hydrogen as an attractive and important energy storage platform by energy utilities; the interest of major global telecoms in fuel cell backup power; and the commercialization of fuel cell electric vehicles (FCEV) by the world’s major automakers. With an increasing number of truly global companies utilizing and investing in fuel cell technology and with the supply chain improvements and technology recognition that will come with the mass manufacture of passenger vehicles, the fuel cell industry is becoming increasingly well-aligned for global success.

## Footnotes

1 http://www.myfcpower.com.
